# Prevalence and Prognostic Significance of Malnutrition in Patients with Type B Aortic Dissection Undergoing Endovascular Repair

**DOI:** 10.31083/j.rcm2507249

**Published:** 2024-07-05

**Authors:** Ting Zhou, Songyuan Luo, Wenhui Lin, Yinghao Sun, Jizhong Wang, Jitao Liu, Yuan Liu, Wenhui Huang, Fan Yang, Jie Li, Jianfang Luo

**Affiliations:** ^1^Department of Cardiology, Guangdong Provincial People’s Hospital (Guangdong Academy of Medical Sciences), Southern Medical University, 510515 Guangzhou, Guangdong, China; ^2^Department of Cardiology, Guangdong Cardiovascular Institute, Guangdong Provincial People's Hospital (Guangdong Academy of Medical Sciences), Southern Medical University, 510515 Guangzhou, Guangdong, China; ^3^School of Medicine, South China University of Technology, 510641 Guangzhou, Guangdong, China; ^4^Department of Emergency and Critical Care Medicine, Guangdong Provincial People's Hospital, Guangdong Academy of Medical Sciences, 510080 Guangzhou, Guangdong, China

**Keywords:** malnutrition, controlling nutritional status score, type B aortic dissection, thoracic endovascular aortic repair, outcomes

## Abstract

**Background:**

Malnutrition is a poor prognostic factor in a wide range of diseases. Nevertheless, there is a lack of data investigating the association between malnutrition and outcomes of patients with type B aortic dissection (TBAD) undergoing thoracic endovascular aortic repair (TEVAR). Therefore, the aim of the present study was to report the prevalence and clinical impact of malnutrition assessed by the controlling nutritional status (CONUT) score in TBAD patients undergoing TEVAR.

**Methods:**

The retrospective study indicated that a total of 881 patients diagnosed with TBAD and treated with TEVAR from January 2010 to December 2017 were categorized into subgroups based on their CONUT score (low ≤5 *vs*. high >5). To assess the correlation between malnutrition and early and follow-up outcomes of TBAD patients, logistic and Cox regression analysis were utilized, incorporating inverse probability weighting.

**Results:**

Malnutrition was present in 20.3% of patients according to the CONUT score. Multivariate logistic regression analysis revealed that pre-operative CONUT score modeled as a continuous variable was an independent risk factor for prolonged intensive care unit stay (odds ratio [OR], 1.09; 95% confidence interval [CI], 1.02–1.17; *p* = 0.015), 30-day death (OR, 1.43; 95% CI, 1.19–1.72; *p* < 0.001), delirium (OR, 1.11; 95% CI, 1.01–1.23; *p* = 0.035) and acute kidney injury (OR, 1.09; 95% CI, 1.01–1.16; *p* = 0.027). During a median follow-up of 70.8 (46.1–90.8) months, 102 (11.8%) patients died (high CONUT group: 21.8% *vs*. low CONUT group: 9.0%; *p* < 0.001). Multivariable Cox proportional-hazards models showed that malnutrition was an independent predictor for follow-up mortality (hazard ratio, 1.68; 95% CI, 1.11–2.53; *p* = 0.014). Results remained consistent across various sensitivity analyses.

**Conclusions:**

Malnutrition assessed by the CONUT score could profoundly affect the early and follow-up prognosis in patients undergoing TEVAR. Routine pre-intervention nutritional evaluation might provide valuable prognostic information.

## 1. Background

Type B aortic dissection (TBAD) is a life-threating vascular event with an elevated risk of serious complications and morbidity [1]. Thoracic endovascular aortic repair (TEVAR) is successful therapy for patients with TBAD, enhances both short- and long-term survival. However, the post-operative death rate remains elevated, especially during long-term follow-up [2, 3]. Therefore, it is essential to promptly recognize risk factors for post-operative morbidity and mortality, and then address reversible risk factors to improve patient outcomes and decrease subsequent costs.

Despite being frequently overlooked, malnutrition is prevalent among patients with aortic diseases and is associated with an unfavorable prognosis [4, 5, 6]. Aortic dissection may trigger inflammation, decrease in appetite, and a catabolic condition, leading to malnutrition [7, 8]. Malnutrition may also accelerate disease progression due to the vicious cycle associated with muscle wasting, reduction in physiological reserves, and degeneration of the aorta medial wall [7, 9]. Malnutrition, as a modifiable risk factor, offers the advantage of allowing timely intervention when compared to other clinical variables.

Scoring systems could be useful to evaluate one’s nutritional status. Numerous screening tools for malnutrition have been developed, but a consensus on the optimal evaluation method has not been reached. One of these assessment tools, controlling nutritional status (CONUT) score, has been widely reported as a simple and efficient method to evaluate nutritional status. It has also been shown to be linked to negative outcomes in various diseases [5, 6, 10, 11]. Despite the significance of nutritional assessment for vascular surgery diseases, there is limited data on the relationship between the nutritional state and the outlook of patients who undergo TEVAR.

## 2. Methods

### 2.1 Study Population

This single-center, retrospective observational study included 992 consecutive patients with TBAD undergoing TEVAR from January 2010 to December 2017. The diagnosis of TBAD was validated through enhanced computed tomography angiography (CTA). The inclusion criteria were the patients of TBAD undergoing TEVAR. Exclusion of patients occurred due to the following factors: (1) blunt traumatic aortic injury, (2) malignant tumor, (3) disorders of connective tissue, (4) prior surgical intervention on the aorta, (5) insufficient data for nutritional assessment (**Supplementary Fig. 1**). The final analysis included the remaining 881 participants. The ethics committee of Guangdong Provincial People’s Hospital (#201807) gave approval for this study and waived the requirement for informed consent.

### 2.2 Definitions and Data Collection

Blood samples were harvested at admission and blood routine examination, lipid profile test, and other laboratory indicators analysis were performed in the central laboratory of the hospital. The CONUT score was created and confirmed as a tool for evaluating the nutritional status of patients admitted to the hospital. It is calculated by adding up the scores of total lymphocytes, albumin level, and total cholesterol levels (**Supplementary Table 1**) [10]. Scores on a scale of 0 to 12, with higher scores indicating a more unfavorable condition. To detect sarcopenia, the skeletal muscle mass index (SMI) was determined using the following formulas: for males, 0.220 multiplied by the body mass index (BMI) and then added to 2.991; for females, 0.141 multiplied by the BMI and then added to 3.377 [12]. Complex TBAD was defined as TBAD accompanied by persistent pain, unresponsive hypertension despite maximum medication, rapid aortic enlargement, malperfusion syndromes, and signs of rupture (such as hemothorax, increasing periaortic and mediastinal hematoma) [1].

### 2.3 Treatment

All patients received with standardized medications and TEVAR treatment following the current guideline and consensus [1, 13]. Patients who had uncomplicated TBAD were subjected to TEVAR if the aortic diameter exceeded 40 mm, primary entry tear diameter >10 mm, false lumen (FL) diameter >22 mm and a patent or partially thrombosed FL [14]. The details of the procedures at our center have been described elsewhere [15]. Briefly, the stent-graft was inserted in a reverse manner through percutaneous femoral artery entry to close the initial tear and the stent-graft size was typically larger by 5% to 10%. When needed, the left subclavian artery (LSA) and/or left common carotid artery (LCCA) were occluded in order to achieve a minimum proximal landing zone of 1.5 centimeters. The method to reconstructing the arch vessels (including chimney or hybrid techniques like TEVAR combined with supra-arch bypass) was determined by the operating surgeon based on the specific characteristics of the aortic pathologies.

### 2.4 Follow-Up and Outcomes

All in-hospital survival patients received clinical and the image of CTA follow-up at 3, 6, 12 months, and subsequently on an annual basis. The assessment of the patient’s state was carried out either by visiting the outpatient clinic or by conducting a telephone interview. The primary outcome were thirty-day death and follow-up mortality. The secondary results included early outcomes that happened during the hospital stay or within 30 days after the procedure. These outcomes encompassed mortality, extended stay in the intensive care unit (ICU), confusion, stroke caused by lack of blood supply to the brain, reduced blood flow to limbs or organs, reduced blood flow to the spinal cord, acute kidney damage, the need for further intervention, and subsequent intervention or stroke during follow-up.

### 2.5 Statistical Analysis

Mean ± standard deviation or median and interquartile range (IQR) are used to express continuous variables based on their distributions and compared using Student’s *t*-test or the Manne-Whitney U test, as appropriate. The presentation of categorical variables is in the form of n (%) and they were compared using either the chi-square test or Fisher’s exact test. To evaluate the associations between patient characteristics and pre-operative nutritional status, Spearman’s rank correlation test was utilized.

The primary predictor was pre-operative CONUT score modeled as a continuous variable. The CONUT score (>5) before surgery was used as a categorical variable in the secondary prediction model. Youden’s index was used to determine the optimal threshold value of the CONUT score (CONUT = 5) for predicting post-operative mortality, which was obtained from the receiver operating characteristic (ROC) curves [16]. High CONUT (>5) was defined as the malnutrition. To examine the connection between the nutritional status before surgery and the initial results, logistic regression models were utilized.

The Kaplan-Meier method was used to present survival data and the log-rank test was employed to compare differences in survival. To evaluate the impact of preoperative nutritional condition on subsequent overall mortality, Cox proportional-hazards regression models were employed. Formal tests were conducted to examine the assumption of proportional hazard, utilizing the techniques outlined by Grambsch and Therneau [17]. There was no indication of any breaches of this assumption. An initial multivariable Cox regression model included demographic characteristics, comorbidities, laboratory tests, and imaging findings. Variables that had a *p* value less than 0.1 in the univariable analysis were included in the multivariable models using a forward stepwise technique. Additionally, to reduce bias and mimic randomization, two propensity-score methods were used to account for potential confounding by characteristics influencing outcomes. A multivariate logistic regression model with covariates was used to estimate individual tendencies for each subject.

The primary analysis used inverse probability of treatment weighting (IPTW) [18]. The stabilized IPTW weight was calculated using the predicted probabilities derived from the propensity-score model in the IPTW analysis. Cox regression models that used the IPTW weights were reported. In the same Cox regression model, we conducted a secondary analysis incorporating the propensity score as an extra covariate.

To further analyze the data, the CONUT score threshold was modified, categorizing it into different levels: normal (0–1), mild malnutrition (2–4), moderate malnutrition (5–8), and severe malnutrition (>8) [10]. R software (version 4.0.3, R Foundation for Statistical Computing, Vienna, Austria) and IBM SPSS 25.0 (SPSS 25 Inc., Armonk, NY, USA) were utilized for conducting all statistical analyses. A significance level of less than 0.05 was deemed significant.

## 3. Results

### 3.1 Clinical Characteristics

**Fig. 1.**
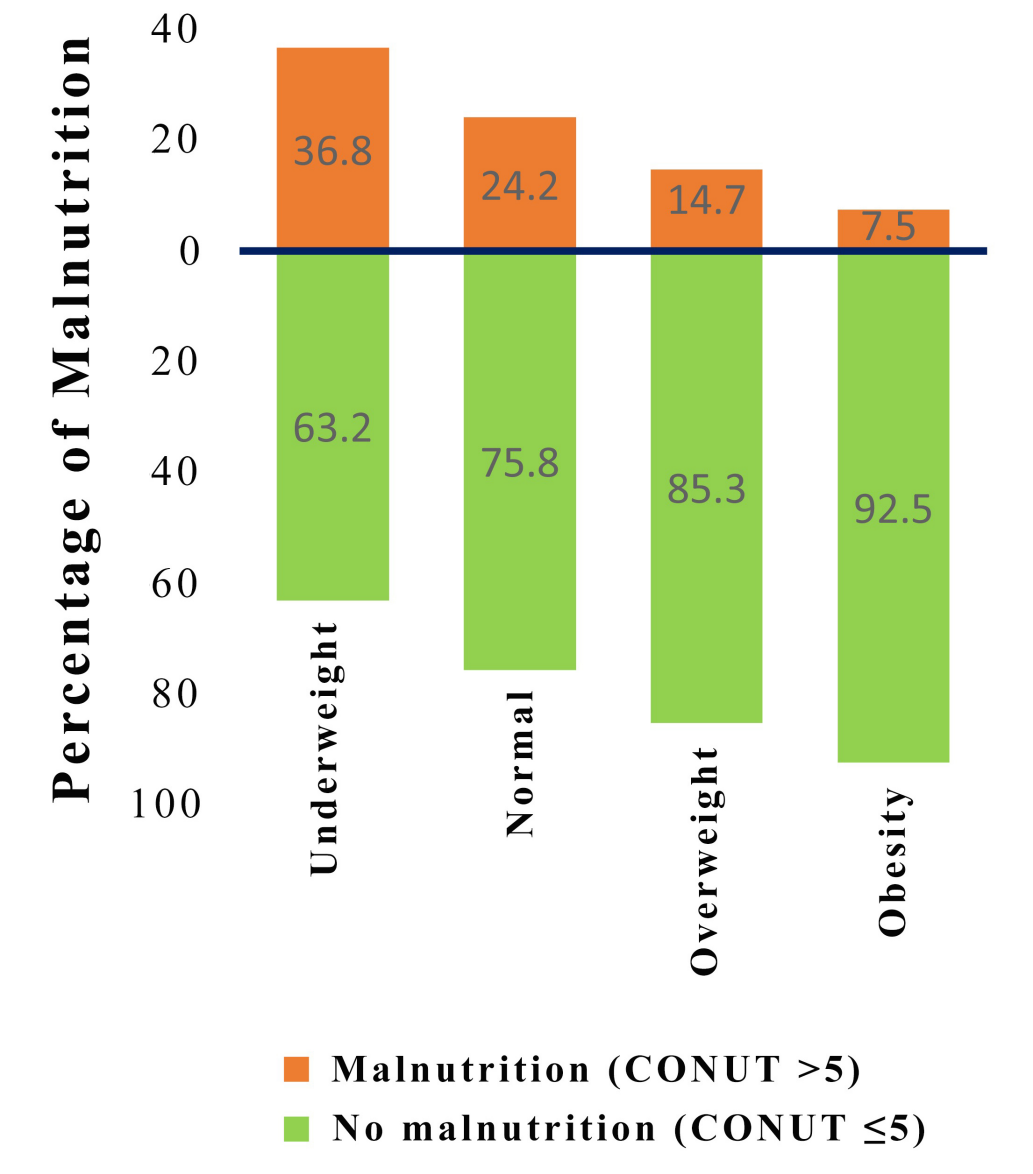
**Distribution of malnutrition by subgroups of patients according to body mass index.** CONUT, controlling nutritional status.

Of the 881 patients enrolled, the average age was 54.2 ± 10.9 years and the majority of the participants (86.6%) were male. The average BMI was 24.5 ± 3.7 ${\mathrm{kg/m}}^{2}$, and the most common comorbid condition was hypertension (84.8%). Based on the optimal CONUT score threshold, patients were divided into two groups: the high CONUT group (>5, n = 702) and the low CONUT group (≤5, n = 179). Additional details of baseline characteristics are presented in Table 1. The prevalence of malnutrition defined as CONUT score greater than 5 was 20.3%. Patients with a BMI below 18.5 ${\mathrm{kg/m}}^{2}$ had the highest rate of malnutrition, accounting for 36.8% (Fig. 1). More than 10% of patients (48/359) suffered malnutrition even in the overweight/obesity group. The distribution of malnutrition did not significantly differ between men and women (19.8% *vs*. 23.7%; *p* = 0.322).

**Table 1.** **Baseline characteristics stratified by controlling nutritional status (CONUT) score**.

Variables		Low CONUT	High CONUT	*p*
		(≤5, n = 702)	(>5, n = 179)	
Age, years		53.6 (10.8)	56.4 (11.2)	0.003
Age >65 years		103 (14.7)	40 (22.3)	0.018
Sex, male		612 (87.2)	151 (84.4)	0.386
BMI, ${\mathrm{kg/m}}^{2}$		24.5 (22.5, 26.9)	23.0 (20.4, 25.1)	<0.001
SMI, ${\mathrm{kg/m}}^{2}$		8.3 (7.7, 8.8)	7.9 (7.4, 8.4)	<0.001
Complicated TBAD		430 (61.3)	104 (58.1)	0.493
Phases of artic dissection				0.004
	Acute	529 (75.4)	131 (73.2)	0.550
	Subacute	109 (15.5)	42 (23.5)	0.012
	Chronic	64 (9.1)	6 (3.4)	0.011
Co-morbidities				
	Hypertension	600 (85.5)	147 (82.1)	0.319
	Coronary artery disease	98 (14.0)	35 (19.6)	0.080
	Diabetes mellitus	45 (6.4)	13 (7.3)	0.809
	Anemia	283 (40.3)	137 (76.5)	<0.001
	Hyperlipoidemia	93 (13.2)	17 (9.5)	0.219
	Stroke	24 (3.4)	6 (3.4)	>0.999
	Abdominal aortic aneurysm	20 (2.8)	8 (4.5)	0.387
Imaging findings				
	MAD, mm	37.9 (34.0, 43.0)	38.0 (34.9, 44.0)	0.496
	MAD >40, mm	244 (34.8)	66 (36.9)	0.659
Extent of the dissection				0.279
	Confined in thoracic aorta	133 (18.9)	41 (22.9)	
	Extended to abdominal aorta	569 (81.1)	138 (77.1)	
False lumen patency				0.259
	Patent false lumen	462 (65.8)	116 (64.8)	
	Partial thrombosis	212 (30.2)	60 (33.5)	
	Complete thrombosis	28 (4.0)	3 (1.7)	
The involvement of visceral arteries		254 (36.2)	46 (25.7)	0.011
The involvement of renal arteries		315 (44.9)	63 (35.2)	0.024
Pericardial effusion		18 (2.6)	17 (9.5)	<0.001
Pleural effusion		274 (39.0)	91 (50.8)	0.005
Liver cyst		96 (13.7)	15 (8.4)	0.075
Renal cyst		151 (21.5)	37 (20.7)	0.887
Laboratory tests				
	White blood cell, ${\mathrm{10}}^{9}$/L	10.5 (8.3–12.7)	9.7 (7.9–13.1)	0.521
	Hemoglobin, g/L	133.4 (122.1–142.0)	119.0 (107.3–129.0)	<0.001
	Lymphocyte, ${\mathrm{10}}^{9}$/L	1.7 (1.3, 2.1)	1.2 (1.0, 1.5)	<0.001
	Albumin, g/dL	34.5 (31.8, 37.0)	27.6 (25.2, 29.2)	<0.001
	Total cholesterol, mg/dL	4.5 (3.9, 5.1)	3.5 (3.1, 4.1)	<0.001
	Triglyceride, mmol/L	1.3 (1.0, 1.7)	1.1 (0.8, 1.5)	<0.001
	LDL-c, mmol/L	2.7 (2.2, 3.2)	2.1 (1.7, 2.6)	<0.001
	D-dimer, µg/mL	2.2 (0.7, 3.9)	2.1 (0.9, 3.8)	0.584
	Creatinine, mg/dL	1.0 (0.8, 1.3)	1.1 (0.9, 1.7)	<0.001
	Creatinine >2 mg/dL	50 (7.1)	37 (20.7)	<0.001
	eGFR, mL/min/1.73 $m^{2}$	83. 6 (62.3, 97.4)	69.0 (43.3, 91.8)	<0.001
	eGFR <60 mL/min/1.73 $m^{2}$	161 (22.9)	73 (40.8)	<0.001
Operative procedure				
	Hybrid	171 (24.4)	40 (22.3)	0.642
	Chimney	142 (20.2)	28 (15.6)	0.200
	Insertion of ≥2 aortic stents	62 (8.8)	25 (14.0)	0.055
Medications at admission				
	Antiplatelet drugs	129 (18.4)	23 (12.8)	0.102
	ACEI	137 (19.5)	38 (21.2)	0.683
	ARB	335 (47.7)	71 (39.7)	0.065
	Beta-blockers	654 (93.2)	168 (93.9)	0.870
	Calcium channel blockers	541 (77.1)	134 (74.9)	0.601

Values are given as mean ± SD, number (percentage) or median (quartiles 1 through 3). BMI, body mass index; SMI, skeletal muscle mass index; TBAD, type B aortic dissection; MAD, maximum aortic diameter; LDL-c, low-density lipoprotein cholesterol; eGFR, estimated glomerular filtration rate; ACEI, angiotensin-converting enzyme inhibitor; ARB, angiotensin receptor blocker; SD, standard deviation.

Individuals belonging to the high CONUT category exhibited a greater tendency towards advanced age, elevated occurrences of subacute individuals, anemia, the engagement of visceral arteries, the engagement of renal arteries, pericardial effusion, pleural effusion, creatinine levels surpassing 2 mg/dL, and an estimated glomerular filtration rate (eGFR) <60 mL/min/1.73 $m^{2}$. Additionally, they exhibited reduced BMI, SMI, lymphocyte count, albumin concentration, total cholesterol (TC) concentration, triglyceride concentration, and low-density lipoprotein cholesterol (LDL-c) concentration. More data on the baseline characteristics were detailed in Table 1.

The CONUT score was significantly associated with age, BMI, SMI, hemoglobin, albumin, lymphocyte, TC, triglyceride, LDL-c, creatinine, and eGFR (*p* < 0.05 for all), as indicated in Table 2. Multivariable logistic regression analysis revealed that anemia (odds ratio [OR], 1.76; 95% confidence interval [CI], 1.07–2.90) and serum albumin (OR, 0.55; 95% CI, 0.50–0.60) were the independent predictors for malnutrition.

**Table 2.** **Spearman correlation between CONUT score and variables**.

Variables	Correlation	*p*
	Coefficient	
Age	0.174	<0.001
Gender	0.049	0.146
Systolic blood pressure	0.008	0.801
Diastolic blood pressure	–0.016	0.639
Body mass index	–0.232	<0.001
Skeletal muscle mass index	–0.228	<0.001
White blood cell count	0.033	0.321
Hemoglobin	–0.411	<0.001
Albumin	–0.779	<0.001
Lymphocyte count	–0.429	<0.001
Total cholesterol	–0.383	<0.001
Triglyceride	–0.192	<0.001
Low-density lipoprotein cholesterol	–0.464	<0.001
D-dimer	0.039	0.250
Creatinine	0.234	<0.001
Estimated glomerular filtration rate	–0.261	<0.001

CONUT, controlling nutritional status.

### 3.2 Early Outcomes

Patients classified in the high CONUT group had significantly higher rates of 30-day prolonged ICU stay, mortality, and post- surgical confusion (*p* < 0.05 for all), However, they exhibited comparable occurrences of stroke, limb and organ ischemia, spinal cord ischemia, and re-intervention (Table 3). Moreover, the prevalence of post-operative acute kidney injury (AKI) was greater in the high CONUT category (30.7%) compared to the low CONUT category (24.5%), although this disparity did not achieve statistical significance.

**Table 3.** **Post-operative outcomes**.

		Low CONUT	High CONUT	*p*
		(≤5, n = 702)	(>5, n = 179)	
Early outcomes				
	Hospital stays, days	13.0 (9.0–18.0)	14.0 (10.0–19.0)	0.157
	Prolonged ICU stay *	192 (27.4)	67 (37.4)	0.008
	Death	12 (1.7)	8 (4.5)	0.043
	Cerebral infarction	19 (2.7)	5 (2.8)	>0.999
	Delirium	59 (8.4)	26 (14.5)	0.013
	Limb ischemia	14 (2.0)	4 (2.2)	0.771
	Visceral ischemia	2 (0.3)	2 (1.1)	0.185
	Spinal cord ischemia	9 (1.3)	3 (1.7)	0.717
	Acute kidney injury	172 (24.5)	55 (30.7)	0.089
	Re-intervention	8 (1.1)	3 (1.7)	0.474
Follow-up outcomes				
	All-cause Mortality	63 (9.0)	39 (21.8)	<0.001
	Re-intervention	37 (5.3)	9 (5.0)	0.896
	Stroke	21 (3.0)	7 (3.9)	0.531

Values are given as number (percentage) or median (quartiles 1 through 3). CONUT, controlling nutritional status; ICU, intensive care unit. *Prolonged ICU stay was defined as intensive care unit stay greater than 72 hours.

Multivariable logistic analyses indicated that the CONUT score, which assessed pre-operative nutritional status, was a significant independent predictor of prolonged ICU stay (OR, 1.09; 95% CI, 1.02–1.17; *p* = 0.015), 30-day death (OR, 1.43; 95% CI, 1.19–1.72; *p* < 0.001), delirium (OR, 1.11; 95% CI, 1.01–1.23; *p* = 0.035) and AKI (OR, 1.09; 95% CI, 1.01–1.16; *p* = 0.027) (Table 4). Likewise, an elevated CONUT score (>5) was found to be linked with a longer duration of stay in the ICU (OR, 1.69; 95% CI, 1.15–2.50; *p* = 0.008), while it did not show any association with other initial unfavorable results (Table 4).

**Table 4.** **Association of CONUT score on early outcomes after multivariable adjustment**.

Variable		CONUT score ^#^		CONUT ≤5 *vs*. CONUT >5		Severe malnutrition (>8) *vs*. normal (≤1)	
		OR (95% CI)	*p*	OR (95% CI)	*p*	OR (95% CI)	*p*
Early outcomes							
	Prolonged ICU stay *	1.09 (1.02–1.17)	0.015	1.69 (1.15–2.50)	0.008	2.03 (0.68–6.10)	0.206
	Thirty-day Death	1.43 (1.19–1.72)	<0.001	2.41 (0.89–6.56)	0.084	31.12 (2.82–343.09)	0.005
	Cerebral infarction	0.99 (0.79–1.23)	0.915	0.97 (0.30–3.12)	0.963	6.00 (0.54–66.42)	0.144
	Delirium	1.11 (1.01–1.23)	0.035	1.67 (0.98–2.85)	0.058	4.31 (1.29–14.39)	0.017
	Limb ischemia	0.93 (0.72–1.19)	0.929	0.76 (0.20–2.88)	0.685	3.15 (0.19–51.81)	0.421
	Spinal cord ischemia	0.96 (0.69–1.34)	0.810	0.71 (0.14–3.65)	0.685	-	0.998
	Acute kidney injury	1.09 (1.01–1.16)	0.027	1.39 (0.91–2.11)	0.124	3.06 (1.16–8.06)	0.024
	Re-intervention	0.86 (0.61–1.23)	0.414	1.22 (0.21–6.93)	0.825	-	0.998
Follow-up outcome							
	Mortality	1.13 (1.05–1.23)	0.002	1.68 (1.11–2.53)	0.014	4.20 (1.46–12.14)	0.008
	Stroke	1.11 (0.91–1.36)	0.290	2.07 (0.75–5.72)	0.162	-	0.952
	Re-intervention	0.90 (0.77–1.06)	0.193	1.08 (0.48–2.43)	0.862	-	0.985

OR, odds ratio; CI, confidence interval; ICU, intensive care unit; CONUT, controlling nutritional status. ^#^CONUT score entered the model as a continuous variable. *Prolonged ICU stay was defined as intensive care unit stay greater than 72 hours.

### 3.3 Survival Analysis

**Fig. 2.**
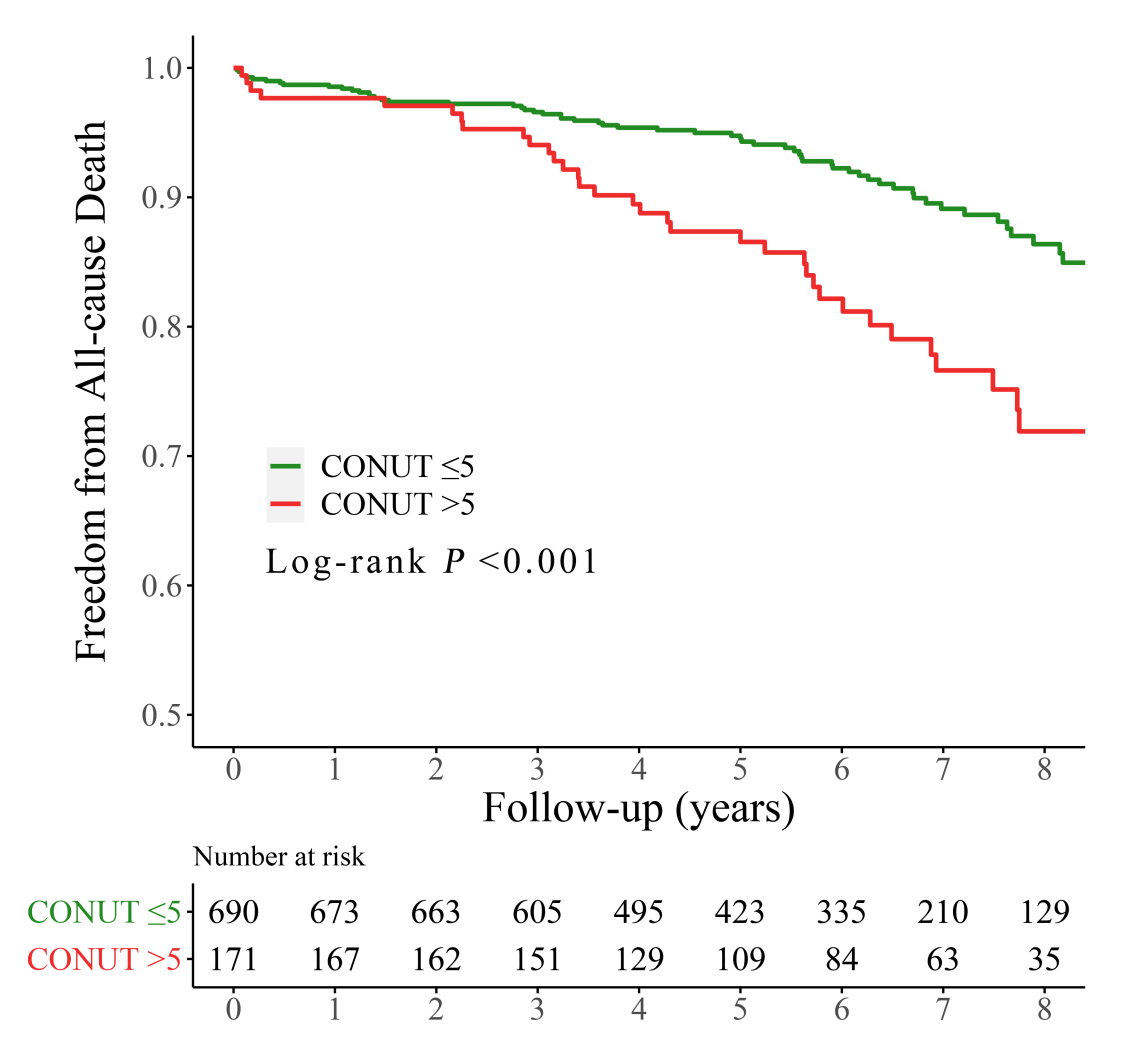
**Kaplan-Meier curves for all-cause mortality by the CONUT score.** CONUT, controlling nutritional status.

Over a period of 70.8 months (with an interquartile range of 46.1–90.8 months), 102 (11.8%) patients died after the procedures, with 39 (21.8%) and 63 (9.0%) patients in the high and low CONUT group, respectively (*p* < 0.001; Table 3). The overall survival rate for follow-up all-cause mortality was considerably greater in the high CONUT category compared to the low CONUT category (21.8% *vs*. 9.0%; log-rank *p* < 0.001; Fig. 2). Furthermore, the occurrence of subsequent stroke and repeat procedure were comparable in both groups (*p* > 0.05 for both; Table 3).

**Fig. 3.**
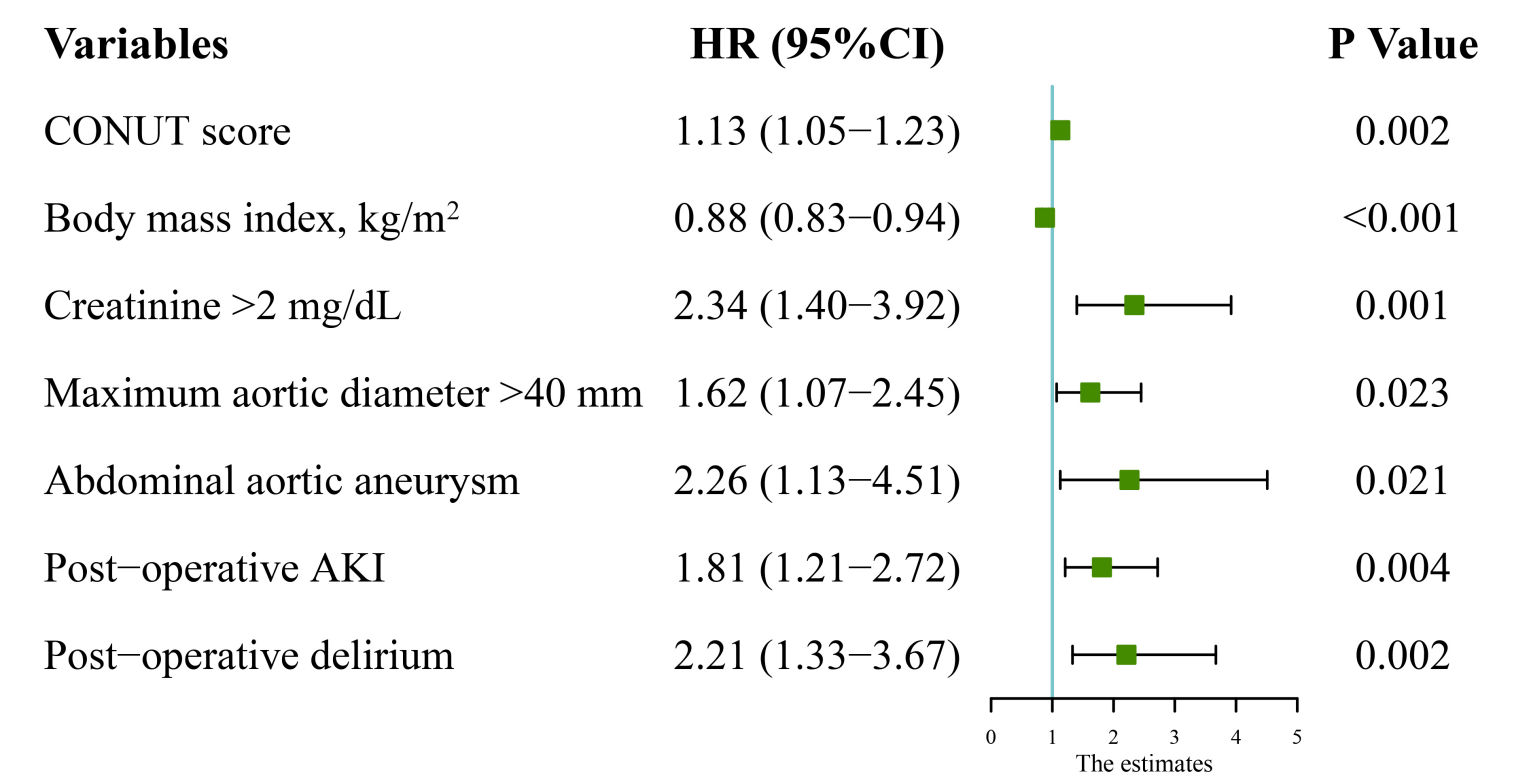
**Multivariate analysis results of follow-up mortality. **HR, hazard ratio; CI, confidence interval; CONUT, controlling nutritional status; AKI, acute kidney injury.

After adjusting for confounding factors (Table 4), Cox multivariate analysis showed that the hazard ratios (HR) for follow-up mortality were 1.13 (95% CI, 1.05–1.23; *p* = 0.002) for CONUT score as a continuous variable. Additional significant factors included BMI (HR, 0.88; 95% CI, 0.83–0.94; *p* < 0.001), creatinine levels >2 mg/dL (HR, 2.34; 95% CI, 1.40–3.92; *p* = 0.001), maximum aortic diameter >40 mm (HR, 1.62; 95% CI, 1.07–2.45; *p* = 0.023), presence of abdominal aortic aneurysm (HR, 2.26; 95% CI, 1.13–4.51; *p* = 0.021), occurrence of post-operative AKI (HR, 1.81; 95% CI, 1.21–2.72; *p* = 0.004) and development of delirium (HR, 2.21; 95% CI, 1.33–3.67; *p* = 0.002) (Fig. 3). Furthermore, the association remained (HR, 1.68; 95% CI, 1.11–2.53; *p* = 0.014) even after including the CONUT as a categorical factor (CONUT score >5; Table 4).

### 3.4 Sensitivity Analysis

In the multivariable analysis using IPTW based on the propensity score, the CONUT score >5 remained as the independent risk factor of follow-up mortality (HR, 1.76; 95% CI, 1.10–2.81; *p* = 0.018; Table 5). A comparable outcome was noted when the propensity score was included in the identical model (HR, 1.68; 95% CI, 1.11–2.53; *p* = 0.014; Table 5). Moreover, there was no correlation between the CONUT score and subsequent re-interventions (*p* = 0.193) or the incidence of stroke (*p* = 0.290).

**Table 5.** **Associations between categorical CONUT score (≤5 *vs*. >5) and follow-up mortality in the crude analysis, multivariable analysis, and propensity-score analyses**.

Analysis		Follow-up mortality	*p*
No. of events/no. of patients at risk (%)			
	CONUT score ≤5	63/702 (9.0)	-
	CONUT score >5	39/179 (21.8)	-
Crude analysis - HR (95% CI)		2.33 (1.56–3.48)	<0.001
Multivariable analysis - HR (95% CI) *		1.68 (1.11–2.53)	0.014
Propensity-score analyses - HR (95% CI)			
	With inverse probability weighting ^#^	1.76 (1.10–2.81)	0.018
	Adjusted for propensity score †	1.68 (1.11–2.53)	0.014

* Shown is the hazard ratio from the multivariable Cox proportional-hazards model adjusting for age >65 years, body mass index, phase of the dissection, complicate aortic dissection, comorbidities (coronary artery disease, diabetes mellitus, hyperlipoidemia, anemia, stroke and abdominal aortic aneurysm), laboratory tests (D-dimer, Creatinine >2 mg/dL and eGFR <60 mL/min/1.73 $m^{2}$), imaging findings (maximum aortic diameter >40 mm, extent of the dissection, false lumen patency, the involvement of visceral arteries, the involvement of renal arteries, pericardial effusion, pleural effusion, liver cyst and renal cyst), operative procedure (hybrid technique, chimney technique, insertion of ≥2 aortic stents), post-operative delirium and acute kidney injury. ^#^Presented here is the primary analysis featuring a hazard ratio derived from the multivariable Cox proportional hazards model, utilizing identical strata and covariates with inverse probability weighting based on the propensity score. †The hazard ratio derived from a multivariable Cox proportional hazards model, incorporating identical strata and covariates, with further adjustment for the propensity score, is presented. CONUT, controlling nutritional status; HR, hazard ratio; CI, confidence interval; eGFR, estimated glomerular filtration rate.

Moreover, the threshold for the CONUT score was modified, and malnutrition was categorized as follows: a CONUT score of 0 to 1 was classified as normal, while scores of 2 to 4, 5 to 8, and 9 to 12 were designated as mild, moderate, and severe malnutrition, respectively [10]. Pre-operative significant undernourishment persisted as a separate forecaster for death within 30 days, delirium after surgery, acute kidney injury after surgery, and mortality during follow-up (*p* < 0.05 for all; see Table 4).

## 4. Discussion

In this study, we discovered that patients with higher CONUT scores faced a heightened likelihood of experiencing an extended stay in the ICU, mortality within 30 days, post-operative delirium and AKI, as well as mortality during the follow-up period. The independent correlation between malnutrition and follow-up mortality remained after modifying the threshold for malnutrition. Propensity-score methods further validated these findings, indicating that CONUT serves as an autonomous and dependable predictor of the initial and prolonged outcomes in TBAD patients who undergo TEVAR.

In our study, the CONUT score classified over 20% of TBAD patients as malnourished, with the greatest percentage found among patients who were underweight (36.8%). Notably, 10.3% of patients with BMI ≥25 ${\mathrm{kg/m}}^{2}$ were malnourished. In Roubín *et al*.’s [10] research on acute coronary syndrome, nearly half (48%) of the patients were categorized as malnutrition. The variation in the CONUT score cut-off value could be responsible for this inconsistency. When we used the same threshold value of 2, above 70% of TBAD patients with overweight/obesity status were stratified as undernutrition. Regardless, these findings emphasized that malnutritional screening should be embedded into routine clinical assessment even in patients with overweight and obesity.

The outcome of our study revealed a correlation between malnourishment and reduced long-term survival, as previously documented in patients who underwent percutaneous coronary intervention [10, 11, 19], coronary artery bypass graft surgery (CABG) [20], and transcatheter aortic valve replacement [21]. Malnutrition is a complicated condition that involves depleted protein stores, a decline in calories, and weakened immune system [22]. The occurrence of adverse events may be induced by a decrease in the ability of underlying fibrinolysis, platelet inhibition, and antioxidant power, along with an increase in blood viscosity [19]. The CONUT score, which is determined by measuring serum albumin, total cholesterol level, and total lymphocyte count, has been confirmed as an effective screening method for malnutrition and is linked to decreased survival rates in conditions such as coronary artery disease [10], peripheral artery disease [5], valvular disease [21] and so on. In addition to reflecting the nutritional status, albumin could also be an indicator of inflammatory responses. The decrease in albumin levels could potentially indicate ongoing damage to the arteries and the advancement of dissection [19, 23]. The number of lymphocytes indicates the immune system of the individual and has been linked to their nutritional condition. The lymphocyte counts decrease, leading to impaired immune defenses [19]. Hypoproteinemia and lymphocytopenia have been demonstrated to be independent risk factors for adverse clinical outcomes of patients with aortic dissection [23, 24]. Additionally, the phenomenon known as the ‘lipid paradox’ or ‘obesity paradox’ has been documented in relation to cardiovascular illness, indicating that individuals with lower lipid levels or BMI may experience unfavorable outcomes [4]. Besides, our study reveals that malnourished patients tend to be older and have a greater burden of diseases, thus confirming a clear association between malnutrition and unfavorable prognosis in TBAD patients.

Multiple research studies have documented a notable association between malnourishment and the emergence of delirium in individuals suffering from acute cardiovascular conditions [25]. Individuals undergoing CABG also showed a comparable correlation [20]. The connection between malnutrition and delirium is still unknown. One possible explanation was that the energy supply to the brain was limited in patients with malnourished, predisposing these subjects to a greater risk of post-operative delirium [25]. Furthermore, the CONUT score can also indicate the level of inflammation, which is considered a significant contributing factor to delirium [26]. In line with these investigations [20, 25], our analysis revealed that the CONUT score independently predicts post-operative delirium in TBAD patients who undergo TEVAR.

Moreover, malnutrition was demonstrated to increase the risk of the occurrence of AKI in hospitalized patients [27]. The current research discovered that CONUT, whether used as a continuous predictor or as a categorical predictor (severe malnutrition [CONUT score >8] *vs.* normal [CONUT score ≤1]), was independently linked to AKI after the procedure, while the optimal cut-off value of 5 did not show any association. AKI was impacted by metabolic alterations and malnourishment due to the spread of an inflammatory process from the kidney to other organ systems [27]. Additionally, patients with malnutrition had a poor baseline renal function in our population, which had been recognized as a well-established predisposing risk factor for AKI [28].

Interestingly, current guidelines and consensus for aortic disease did not emphasize the management of patient’s nutritional status. Nevertheless, our study revealed that malnourishment was a prevalent and significant concern among TBAD patients who underwent TEVAR. Identifying malnutrition in individuals with TBAD could help identify patients who are at a heightened risk of negative clinical outcomes. These patients may benefit from personalized prevention strategies involving nutritional supplements, which can enhance their prognosis. It was anticipated that the diverse approaches, such as the use of oral nutritional supplements, enrichment of food/fluid, counseling on dietary habits, and educational interventions, would be able to alleviate the patients’ malnutrition [29]. Nevertheless, clinicians were supposed to balance the risk of delay in intervention to provide a period of pre-operative nutritional supplement to reduce risk associated with immediate surgery especially in complicated and malnourished TBAD subjects. Careful nutritional assessment and effective management were indispensable for patients with uncomplicated TBAD patients. Furthermore, it is important to maintain nutritional interventions even after being released from the hospital in order to ensure the restoration of a healthy nutritional condition.

This study is subject to several potential constraints. First, it is a retrospective, observational study in a single-center, and therefore subject to selection bias. Patients were consecutively recruited, and propensity score techniques were utilized to alleviate these biases. Second, the examination of the influence of nutritional status dynamics on unfavorable clinical occurrences throughout the monitoring period was not conducted. Third, the potential advantages of nutritional supplements on the clinical results of individuals with TBAD were not investigated. Future studies were expected to confirm our conclusion and establish detailed management strategies of malnutrition for TBAD patients.

## 5. Conclusions

In this study, the prevalence of malnutrition, assessed by CONUT score, is high in TBAD patients undergoing TEVAR and could have a significant impact on their early and follow-up results. Pre-operative nutritional assessment followed by prompt intervention and ongoing multidisciplinary care, may enhance the prognosis of patients.

## Data Availability

The datasets used and/or analyzed during the current study are de-identified and available from the corresponding author on reasonable request.

## Abbreviations

TBAD, type B aortic dissection; TEVAR, thoracic endovascular aortic repair; CONUT, controlling nutritional status; SMI, skeletal muscle mass index; BMI, body mass index; LSA, left subclavian artery; LCCA, left common carotid artery; IPTW, inverse probability of treatment weighting.

## Supplementary Material

Supplementary material associated with this article can be found, in the online version, at https://doi.org/10.31083/j.rcm2507249.
