# Supplementary material for: Prevalence and Prognostic Significance of Malnutrition in Patients with Type B Aortic Dissection Undergoing Endovascular Repair
Source: Rev Cardiovasc Med. 2024 Jul 5;25(7):249. doi: 10.31083/j.rcm2507249 (PMC11317338; doi:10.31083/j.rcm2507249)
Supplement: Supplementary file 1 [file 2153-8174-25-7-249-s1.pdf]

Supplementary Fig. 1

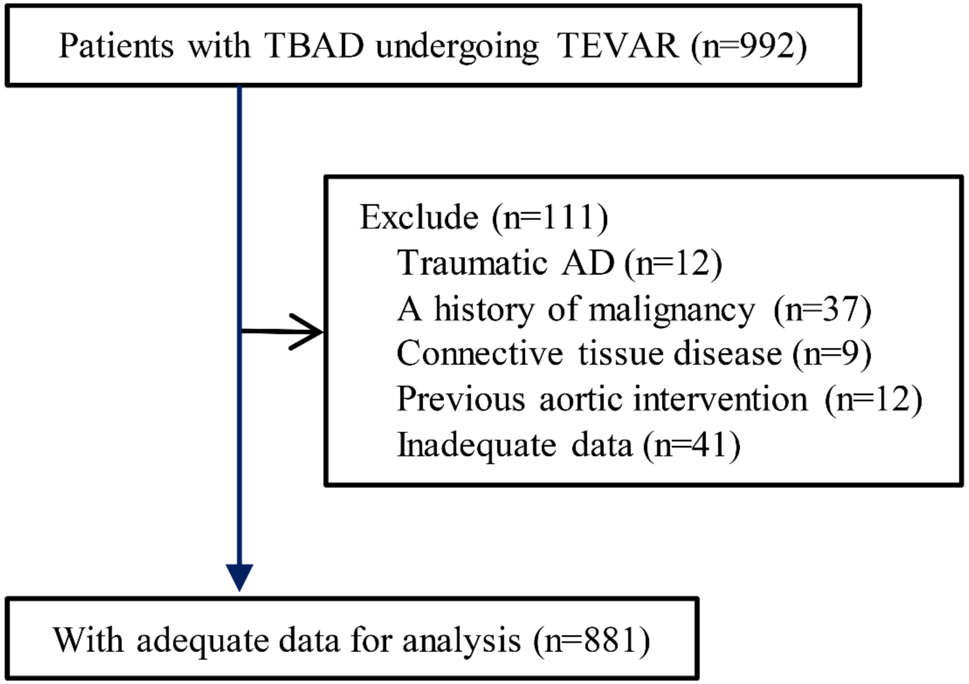

Supplementary Table 1 Controlling nutritional status (CONUT) scores

	Score			
	Normal	Low risk	Moderate risk	Severe risk
Serum albumin, g/dL	≥3.5	3.0-3.49	2.50-2.99	< 2.5
Albumin score	0	2	4	6
Total cholesterol, mg/dL	≥180	140-179	100-139	< 100
Cholesterol score	0	1	2	3
Lymphocytes, count/μL	≥1600	1200-1599	800-1199	< 800
Lymphocyte score	0	1	2	3
Total score	0-1	2-4	5-8	9-12
